# Interactions of pathological proteins in neurodegenerative diseases

**DOI:** 10.1007/s00401-017-1709-7

**Published:** 2017-04-11

**Authors:** Tara L. Spires-Jones, Johannes Attems, Dietmar Rudolf Thal

**Affiliations:** 1 Centre for Dementia Prevention, and Euan MacDonald Centre for Motor Neurone Disease, The University of Edinburgh Centre for Cognitive and Neural Systems, 1 George Square, Edinburgh, EH8 9JZ UK; 2 Institute of Neuroscience, Newcastle University, Campus for Ageing and Vitality, Newcastle upon Tyne, NE4 5PL UK; 3 Departement Neurowetenschappen, Katholieke Universiteit Leuven, Herestraat 49, 3000 Leuven, Belgium; 4 Departement Pathologische Ontleedkunde, UZ Leuven, Herestraat 49, 3000 Leuven, Belgium

**Keywords:** Alzheimer’s disease, Amyotrophic lateral sclerosis, Frontotemporal lobar degeneration, Lewy body, Amyloid beta, Tau, Synuclein, TDP-43

## Abstract

Neurodegenerative diseases such as Alzheimer’s disease (AD), frontotemporal lobar degeneration (FTD), Lewy body disease (LBD), Parkinson’s disease (PD), and amyotrophic lateral sclerosis (ALS) have in common that protein aggregates represent pathological hallmark lesions. Amyloid β-protein, τ-protein, α-synuclein, and TDP-43 are the most frequently aggregated proteins in these disorders. Although they are assumed to form disease-characteristic aggregates, such as amyloid plaques and neurofibrillary tangles in AD or Lewy bodies in LBD/PD, they are not restricted to these clinical presentations. They also occur in non-diseased individuals and can co-exist in the same brain without or with a clinical picture of a distinct dementing or movement disorder. In this review, we discuss the co-existence of these pathologies and potential additive effects in the human brain as well as related functional findings on cross-seeding and molecular interactions between these aggregates/proteins. We conclude that there is evidence for interactions at the molecular level as well as for additive effects on brain damage by multiple pathologies occurring in different functionally important neurons. Based upon this information, we hypothesize a cascade of events that may explain general mechanisms in the development of neurodegenerative disorders: (1) distinct lesions are a prerequisite for the development of a distinct disease (e.g., primary age-related tauopathy for AD), (2) disease-specific pathogenic events further trigger the development of a specific disease (e.g., Aβ aggregation in AD that exaggerate further Aβ and AD-related τ pathology), (3) the symptomatic disease manifests, and (4) neurodegenerative co-pathologies may be either purely coincidental or (more likely) have influence on the disease development and/or its clinical presentation.

## Introduction

Neurodegenerative disorders are characterized by protein aggregates, the importance of which in inducing neuronal toxicity has been hotly debated. Pathological forms of proteins also appear to spread through the brain in characteristic patterns in most neurodegenerative diseases, with particularly robust evidence for the spread of amyloid beta (Aβ), tau (τ), and α-synuclein (α-syn) [43, 73]. Although each neurodegenerative disorder exhibits accumulation of specific characteristic protein aggregates, there are also many cases that exhibit aggregation of multiple pathological proteins. Studies in model systems including transgenic animals and cell culture support several potential types of interactions between different pathological proteins involved in neurodegeneration including cross-seeding of aggregates and pathological changes in one protein initiating mislocalization and post-translational modifications of another. Here, we will examine the neuropathological co-occurrence of pathologies in human autopsy cases and discuss potential mechanistic links between these diverse proteins in disease pathogenesis. We will focus on the pathological proteins for which there is the clearest evidence of both co-existence in human cases and biological evidence for potential mechanistic interactions. Table 1 summarizes the neurodegenerative diseases we will cover and the protein aggregates most commonly observed in the brains of people with those diagnoses. The co-occurrence of cerebrovascular pathology and protein aggregates is discussed in the other article of this cluster [77]. Here, our aim is to provide an updated view about the co-occurrence of different protein aggregates in neurodegeneration and their possible mutual interactions which should further our understanding of the biology of neurodegenerative disorders.

**Table 1:** Pathological aggregates in neurodegenerative diseases

Disease	Proteins that aggregate	Location of aggregates	Brain region(s) affected	Common co-pathologies (% cases where observed)
AD	Amyloid beta (Aβ)—plaques and cerebral amyloid angiopathy (CAA) τ-Protein—neurofibrillary tangles (NFTs), neuropil threads (NTs)	Extracellular with the neuropil (plaques), tunica media and at the basement membrane of cerebral and leptomeningeal blood vessels (CAA) Intracellular within the perikaryon and neuritic cytoplasm of neurons (NFTs, NTs)	Plaques spread from neocortices to the allocortex and down through the basal ganglia, diencephalon, brain stem and cerebellum NFTs spread from transentorhinal cortex into hippocampus and the neocortex	TDP-43 cytoplasmic inclusions (over 50% of cases) [71] α-Syn Lewy neurites and LB (over 40% of cases) [150]
Tauopathies (FTLD-tau, Pick’s disease, argyrophilic grain disease, frontotemporal dementia with chromosome 17 mutation, NFT-predominant dementia, PSP, CBD, PART)	τ-Protein—NFTs, NTs, Pick bodies, coiled bodies, astrocytic plaques, tufted astrocytes, grains, ballooned neurons	Cytoplasmic (neurons and glia)	Frontal and temporal cortex, basal ganglia, and brain stem	except for argyrophilic grain disease which frequently co-occurs with AD pathology, most cases do not have significant co-pathologies
Synucleinopathies (Parkinson’s/DLB/MSA)	α-Syn—LB, Lewy neurites (DLB/PD), glial cytoplasmic inclusions (Papp-Lantos bodies, MSA)	Cytoplasmic [neurons—PD, DLB; oligodendrocytes (Papp-Lantos bodies)—MSA)]; nuclear (neurons—MSA)	Substantia nigra, cortex. In PD, pathology begins in brainstem and spreads up through midbrain to cortex. In DLB, pathology begins in cortex and spreads “down” MSA-P (striato-nigral degeneration with parkinsonism (SDN)): lesions mainly in the striatum and the substantia nigra; gray and white matter pathology MSA-C (olivo-ponto-cerebellar atrophy, OPCA): lesions mainly in the pons, medulla oblongata, and the cerebellum; gray and white matter pathology	Aβ pathology (over 80% of cases) [61] NFT and NT at Braak stage >II (over 50% of cases) [61] TDP-43 pathology (over 30%) [95]
Amyotrophic lateral sclerosis (ALS)	TDP-43, FUS, ubiquilin, C9orf72 translated intronic repeat, SOD1	Cytoplasmic, nuclear (neurons and oligodendrocytes)	Motor cortex, spinal cord, other cortical regions	Neurofibrillary tangles (78%), atherosclerosis (35%); β-amyloid (35%); tauopathy/tau inclusions (17%), Lewy body formation (11%), microinfarcts (7%), and α-syn (4%) [24] τ- and α-syn pathology in cases of the Kii-Peninsula variant in the temporal lobe, striatum brain stem, cerebellum [82, 102]
FTLD-TDP, FTLD-FUS	TDP-43, FUS, ubiquilin, C9orf72 translated intronic repeat	Cytoplasmic, nuclear inclusions in neurons and glial cells	Frontotemporal neocortex and medial temporal lobe	

## Aβ and τ—interactions of the classic hallmarks of Alzheimer’s disease (AD)

AD is the most common dementing disorder seen in 50–60% of dementia cases. Neuropathologically, AD is characterized by dramatic atrophy of the gray matter and accumulation of amyloid plaques and neurofibrillary tangles (NFTs) [60]. Amyloid plaques consist of extracellular aggregates of Aβ [93], which is a cleavage product derived from the amyloid precursor protein (APP) by β- and γ-secretase cleavage [53]. The same Aβ peptide also occurs in AD-related vascular deposits known as cerebral amyloid angiopathy (CAA) [7]. Aβ-plaques as well as vascular Aβ deposits first occur in neocortical areas and then expand into further brain regions in a distinct hierarchical sequence [141, 143].

### Age-related accumulation of Aβ and τ

**Fig. 1:**
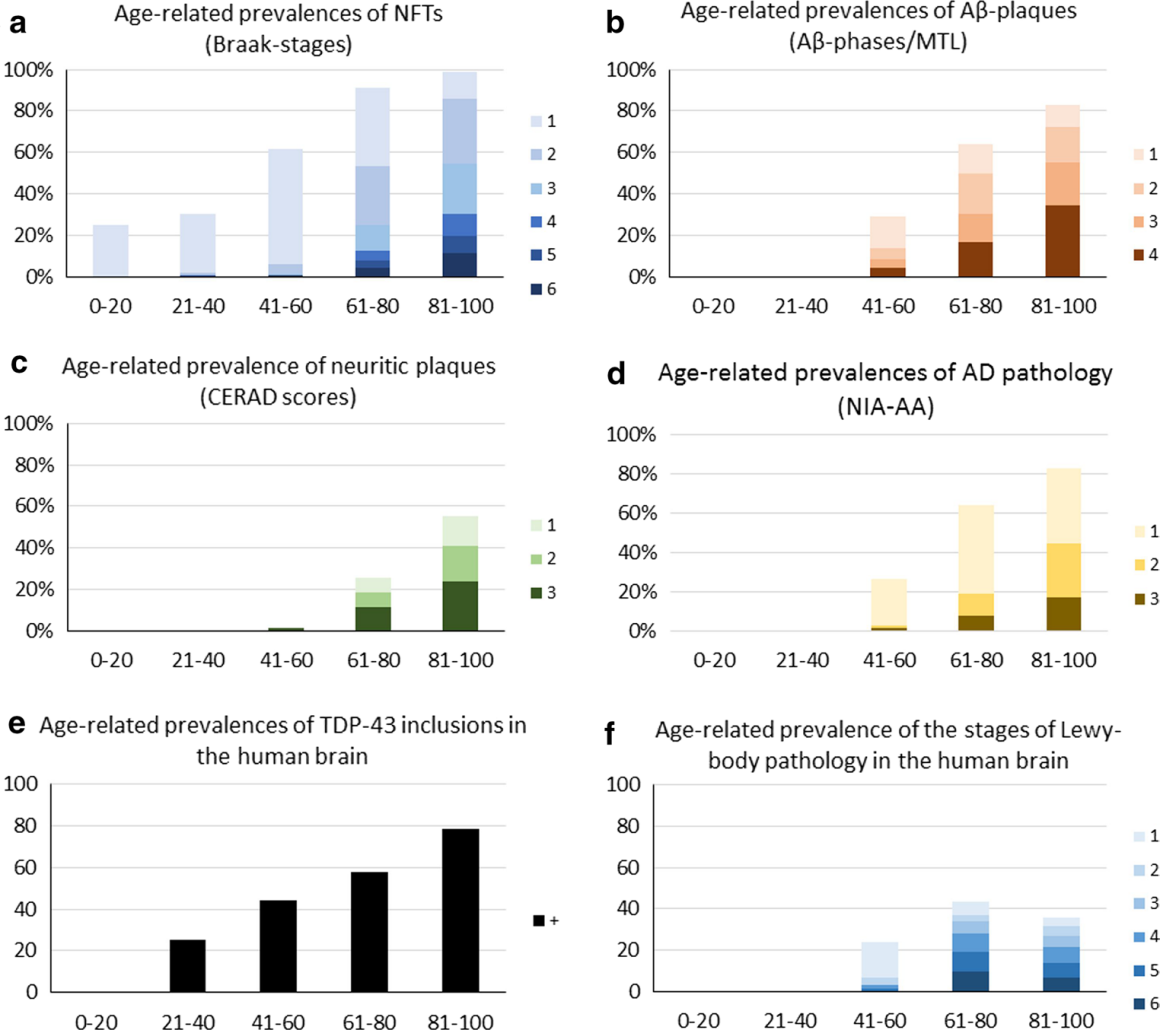
Pathological proteins accumulate with increasing age as measured in large post-mortem studies. Age-related prevalence of NFTs (**a**, *n* = 1310), Aβ plaques (**b**, *n* = 849), neuritic plaques (**c**, *n* = 662), AD pathology as defined by NIA-AA (**d**, *n* = 662), TDP-43 pathology (**e**, *n* = 147), and Lewy body pathology (**f**, *n* = 582) (reevaluation of previously published cases by the authors (JA, DRT) [95, 139, 146, 147]). The prevalence for NFTs is provided separately for each Braak-NFT stage (I–VI) [12] (**a**) and that for Aβ plaques separately for each phase of Aβ-plaque deposition as determined in medial temporal lobe sections (*1*–*4*) [144] corresponding with the plaques extension all over the entire brain [143]. The frequency of neuritic plaques (**c**) is shown separately for each CERAD score (*1*–*3*) [98]. The neuritic plaques were detected in these cases by Gallyas silver staining or immunohistochemistry against abnormal phosphorylated τ-protein (AT-8) [142, 146]. The degree of AD pathology has been assessed in accordance with the current neuropathological criteria for the description of AD pathology as published by the National Institute of Aging and the Alzheimer Association [low (1)—high (3)] [60] (**d**). The prevalence of TDP-43 aggregates was assessed dichotomously on the basis of an immunostaining with an antibody against phosphorylated TDP-43 (presence = 1) (**e**). The prevalence of LB pathology was assessed by classifying the LB pathology according to Braak et al. (*1*–*6*) [14]. Prevalences were provided separately for each stage (**f**). In contrast, to τ, Aβ, and TDP-43 pathology, the prevalence of LB pathology decreases after a peek in the age group 61–80 years probably indicating a life expectancy limiting effect of LB pathology

Age is the most important risk factor for Alzheimer’s disease, and for most sporadic, non-inherited neurodegenerative disorders. However, the question of why age is associated with increased risk for neurodegeneration remains unclear. We have examined pathological cohorts at different ages to characterize pathological accumulations. Aβ deposits are observed as early as 11–20 years of age, and their frequency and phase of distribution increases with increasing age. At 80 years of age, approx. 80% of all autopsy cases show Aβ plaques (see also Fig. 1b), whereas approx. 20% do not develop any amyloid plaques up to the age of 100 years [15]. Symptomatic AD seen in the old age groups exhibits a prevalence ranging from 11% in the age group over 64 years to 32% in the age group over 84 years [3]. In parallel with the τ and Aβ pathology, neuritic plaques also become more prevalent with aging. In the cohort of cases covered in Fig. 1c, they were first seen between 41–60 years of age and reached approximately 55% prevalence in the age group of 81–100 years. Amyloid pathology alone is not sufficient to cause symptomatic Alzheimer’s disease, and many non-demented people show early phases of plaque pathology with aging.

NFTs represent neuronal cytoplasmic aggregates of abnormal τ-protein that form paired helical filaments [48]. The first step in the formation of these aggregates is the abnormal phosphorylation of the τ-protein, the dissociation of τ from axonal microtubules, and its aberrant accumulation in the somatic cytoplasm and dendrites. Somatodendritic accumulations of phosphorylated τ before fibrils are formed and are called “pretangles” [2, 9]. NFTs consisting of paired helical filaments of τ develop after (and presumably directly from) pretangles and correlate with neuronal degeneration. NFTs remain as ghost tangles even after the death of neurons, but the amount of neuronal death in AD exceeds the amount of NFT formation, indicating that NFTs are not necessary for cell death [9, 11, 45]. In the human brain, silver-stainable NFTs start to develop in the transentorhinal cortex and in a few subcortical brain stem nuclei (locus coeruleus, raphe nuclei, basal nucleus of Meynert) [13, 15]. From the transentorhinal cortex, NFT pathology spreads into the entorhinal region and then further into other brain regions in a distinct hierarchical sequence that is different from that for Aβ-plaques [12]. The τ-protein occurs in the human brain in six isoforms, which have a characteristic microtubule-binding repeat region [44]. Three τ isoforms have 3 repeats in the repeat region, whereas the other three isoforms have 4 repeats. In AD, both 3- and 4-repeat isoforms are detected in the τ-aggregates [44].

As with Aβ, the prevalence of τ pathology increases with age but can be detected in surprisingly young people. The earliest signs of an abnormal accumulation/aggregation have been found in a 6-year-old boy [13] in the locus coeruleus (the brainstem nucleus that represents the noradrenergic center of the brain) exhibiting non-fibrillar abnormal phosphorylated τ-protein. These first τ-accumulations were pretangles. Pretangles were seen in other brainstem and subcortical nuclei, namely the raphe nuclei (serotonergic brain stem nuclei), and the basal nucleus of Meynert (cholinergic nuclei of the brain) at later ages. At 40 years of age, most human individuals showed brainstem τ pathology [8, 15]. The first NFTs consisting of paired helical filaments were seen in individuals between 10 and 20 years of age, but the question of whether these NFTs are already associated with AD or if they are unrelated to AD remains controversial [15, 28]. However, with advancing age, NFT-τ pathology becomes more prevalent and higher Braak-NFT stages also become more prevalent. In people over 91 years of age, nearly every individual shows at least Braak-NFT stage 1 [15] (see also Fig. 1a).

Neurofibrillary tangle pathology is not exclusively found in association with Aβ deposition as evidenced by the disease primary age-related tauopathy (PART). PART is characterized by the presence of neuronal τ-protein pathology in the form of neurofibrillary tangles (NFT) and neuropil threads (NT) without (definite PART) or with only limited (possible PART) parenchymal amyloid-β (Aβ) depositions (up to phase 2 according to Thal et al. [143]) [28]. In addition, any other primary tauopathy (such as PSP, CBD, and AGD, etc.) has to be excluded. PART is frequent in old age (up to 40%) and is likely to represent the neuropathological correlate of a condition termed “suspected non-AD pathophysiology” (SNAP), which refers to individuals with temporal lobe atrophy on MRI and high CSF τ but no evidence for cerebral Aβ depositions [64]. SNAP is usually seen in cognitively normal elderly but may also be present in mildly impaired individuals. While the neuroanatomical progression of NFT/NT in PART follows AD-associated NFT-Braak stages [28], the latter do not exceed stage IV in PART and NFT-Braak stages V/VI are indeed only seen in the presence of Aβ pathology.

As PART is a rather novel entity, little is known about the co-occurrence of PART and other neurodegenerative diseases. However, in the Newcastle Brain Tissue Resource (NBTR), 12% of LBD cases show additional PART and in 45% of PART cases, pathological lesions typically associated with other diseases were present (e.g., ALS, stroke, multiple system atrophy), while 55% of all PART cases showed no signs of neurological disease during life and no or only very mild age-associated pathologies at post-mortem examination in addition to PART.

### Mechanistic links between Aβ and τ

Despite the existence of “pure” or “primary” tauopathies, it is clear that Aβ and τ pathologies are related, at least in AD. The link between Aβ and τ pathologies is clearly evidenced in their convergence in the AD brain as neuritic plaques, in which Aβ aggregates are associated with dystrophic neurites, which most frequently exhibit neurofibrillary lesions consisting of aggregates of abnormal phosphorylated τ-protein [32]. Here, intracellular τ in dystrophic neurites together with extracellular Aβ constitute one single pathological lesion, i.e., the neuritic plaque, which is a conditio sine qua non for the neuropathological diagnosis of symptomatic AD. Although plaque and tangle pathologies begin in different brain regions and show different progression pattern (Table 1; Fig. 1), a recent PET imaging study found some convergence zones of Aβ and τ pathologies, particularly in the inferior-lateral temporal lobe, which hints at physical, perhaps network-distributed interactions of these pathologies in disease progression [123].

Further evidence strongly implicating a mechanistic link between Aβ and τ is the genetics of familial AD (fAD), which clearly implicates changes in Aβ processing not only in plaque formation but also in causing τ pathology. fAD mutations have been found in the APP gene, which codes for the amyloid precursor protein (APP) that is cleaved to form Aβ, and also in the presenilin 1 and 2 genes (PSEN1, PSEN2) [42, 120, 130, 131], which form parts of the γ-secretase enzyme responsible for one of the essential proteolytic cleavages of APP to form Aβ [161]. Pathogenic mutations in these genes lead to an increased production of Aβ and to the development of AD. Neuropathologically, these cases show Aβ and τ aggregates similar to those observed in sporadic AD cases. In further support of Aβ generation being key to initiating AD, other mutations in the APP gene that preclude Aβ production have been shown to be protective [69]. Conversely, mutations in the *MAPT* gene encoding τ do not cause fAD, but can lead to neurodegenerative tauopathies which often include Parkinsonian symptoms [59]. Here, τ pathology occurs in neurons, astrocytes, and oligodendrocytes in a FTLD-tau-like pattern, sometimes producing Pick bodies. Co-occurrence of other neurodegenerative features except for age-related amounts are uncommon. The pattern in FTLD-tau cases with τ-mutations is very variable [40]. The *MAPT* gene occurs on a part of chromosome 17 that has two major haplotypes called H1 and H2 due to an ancient inversion of a 900 kb region [132], and these haplotypes have been linked to the risk of several diseases. H1 haplotype is associated with increased risk of the tauopathies progressive supranuclear palsy (PSP) and corticobasal degeneration (CBD) and more recently to the risk of developing late-onset AD (LOAD) in people who do not carry the well-established LOAD risk gene APOE4 [110]. A relatively recently discovered mutation in *MAPT*, A152T, was discovered in a PSP patient and was subsequently found to be associated with increased risk of FTD, PSP, CBD, and AD [27].

Along with the co-occurrence of Aβ and τ pathologies in AD brain, and the human genetic data strongly suggesting that increased production of Aβ may trigger τ pathology, data in model systems also support a series of complex interactions between Aβ and τ. Mouse models have been developed, which exhibit plaques due to overexpression of fAD-associated mutant proteins and τ pathology due to overexpression of FTD-associated mutant τ. Generally, the results of these studies support the role of Aβ in exacerbating τ pathology. For example, crossing Tg2576 mice which develop Aβ pathology and JNPL3 tauopathy mice exacerbated the τ pathology [87]. In the 3xTg mice, which similarly have both Aβ and τ pathology, lowering Aβ levels causes a reduction in τ pathology, but conversely lowering τ levels does not reduce Aβ pathology [108, 157], again arguing that Aβ is upstream of τ pathology. In further support of this idea, mice that expresses human oligomeric Aβ and human τ eventually develop NFT pathology even in the absence of tauopathy-associated mutations in the MAPT gene [152]. Moreover, in cell culture experiments, applying exogenous Aβ to mouse primary neurons instigates τ phosphorylation and mislocalization to dendrites [163], and in human stem cell-derived neuronal cultures, overexpressing fAD mutations in APP and PS1 causes both plaque and tangle pathology [21]. Most of the evidence from genetics, mouse models, and cells indicates that Aβ drives τ pathology, but there are examples of surprising reciprocal interactions. For example, in the APP/PS1 mouse model, plaques are significantly larger in mice expressing either human P301L mutant [114] or wild-type human τ [65]. Not only does Aβ appear to somehow instigate τ pathology, it may also gate the spread of τ pathology through the brain. A recent cross-sectional PET study examining both Aβ and τ indicates this potential effect with hints that Aβ pathology is necessary to start the spread of τ pathology out of the medial temporal lobe [123]. These imaging data accurately reflect neuropathological findings as τ pathology in the absences of Aβ (i.e., PART) does not exceed NFT-Braak stage IV, while NFT-Braak stages V/VI are only seen in the presence of Aβ further indicating an aggravating effect of Aβ on τ pathology. Similar to findings in the human brain, in rTgTauEC mice expressing FTD mutant τ restricted to the entorhinal cortex, the presence of plaques (generated by crossing the rTgTauEC line with APP/PS1 mice) accelerates the spread of τ through neural circuits [114]. However, while Aβ may aggravate τ pathology, it is not a prerequisite for the mere development of τ pathology and hence Aβ must not be regarded as a mandatory upstream event in the development of τ pathology. The existence of non-AD tauopathies such as PSP, Pick’s disease, and CBD strongly support the point of view that Aβ is not required to develop a tauopathy that cases neurological/psychiatric symptoms.

**Fig. 2:**
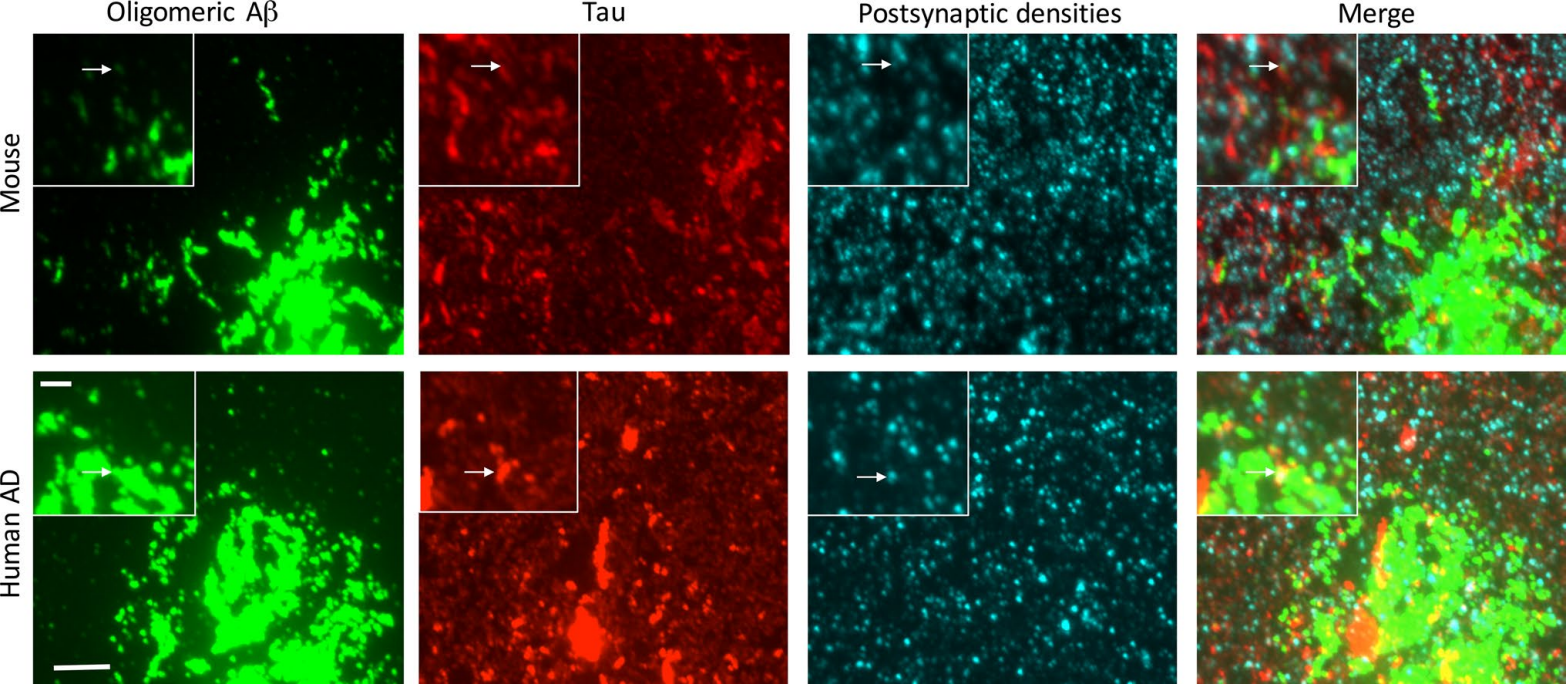
Co-aggregation of pathological proteins in synapses may contribute to neurodegeneration. Synaptic toxicity of pathological proteins is thought to be one of the driving forces in several neurodegenerative diseases. In both a mouse model expressing fAD mutations in APP and PS1 and human wild-type τ ([65], *top*) and human AD brain (*bottom*), we observe colocalization of Aβ and τ at some synapses using the array tomography technique (*arrows*). *Scale bars* represent 5 µm in large panel and 2 µm in *inset*

While pathological lesions of Aβ are mainly extracellular and τ lesions are intracellular, there are places in the brain where the soluble forms of these proteins, which are now thought to be the more toxic, bioactive species, may interact. One such important point of convergence is the synapse. Several factors indicate that synapses are key players in AD pathogenesis: (1) synapse dysfunction and loss occur early in the disease process (2) synapse loss is the strongest pathological correlate of cognitive decline, and (3) synaptic connections appear to be the point of spread of pathological proteins through neural circuits. A large body of literature shows that pathological soluble forms of Aβ and τ can separately induce synaptic dysfunction and synapse loss, and more recently, evidence has begun to accumulate linking Aβ and τ in synapse degeneration [128]. Removing endogenous mouse τ protects mice against some Aβ-mediated synaptic phenotypes such as seizure activity, synaptotoxicity, and long-term potentiation deficits [63, 119, 124], and the synapse loss observed with exogenous Aβ application to cultured neurons is associated with local accumulations of phosphorylated τ in dendrites [163]. Aβ and τ were also shown with immuno-electron microscopy to co-accumulate in postsynaptic terminals in 3xTg AD mice [136], although due to the limitations of electron microscopy, this was in a limited number of animals and was not quantified. We are using the array tomography technique to allow higher throughput quantitative imaging of synapses than EM and to overcome the *z*-resolution limit of light microscopy and accurately study the colocalization of pathological proteins of synapses in mouse and human brain with immunofluorescence [78, 97]. These studies indicate that these proteins do co-occur in a subset of synapses in both mouse and human brain (Fig. 2). Our recent work in a novel mouse model indicates that there is not a strict dose–response relationship in the interactions of Aβ and τ in synapse loss as addition of wild-type human τ in APP/PS1 mice with plaque-associated synapse loss does not exacerbate synapse loss [65]. Future work with temporal control over τ reduction in these plaque-bearing mice will determine whether τ is necessary for Aβ-mediated synaptic phenotypes. Another potential mechanistic interaction at the synapse between proteins involved in different diseases is the role of cellular prion protein (PrPc) in Aβ-mediated synapse degeneration. PrPc becomes misfolded, aggregated, and toxic in Prion diseases such as variant Creutzfeldt–Jakob disease. In addition, PrPc has been proposed as a co-receptor with mGluR5 for oligomeric Aβ at synapses where it activates Fyn kinase and causes impaired synaptic plasticity [57, 85, 151]. This is a potentially very interesting cascade in light of the role of Fyn kinase in mediating τ toxicity at synapses, thus linking three pathological proteins in the same synaptotoxic cascade, at least in mouse models.

## Overlap of α-syn with other pathologies

Synucleinopathies encompass Lewy body diseases (LBD) and multiple system atrophy (MSA). The latter are characterized by glial α-syn aggregations in the form of glial cytoplasmic inclusions (Papp-Lantos bodies) while the neuropathological hallmark lesions of LBD are α-syn aggregates in neuronal cell somata and neuronal cell processes termed Lewy bodies (LB) and Lewy neurites (LN), respectively. LBD encompass Parkinson’s disease (PD), Parkinson’s disease dementia (PDD), and dementia with LB (DLB). While PD is primarily a movement disorder clinically characterized by the presence of extrapyramidal symptoms (EPS), clinical core features of PDD and DLB include fluctuating cognition and visual hallucinations [96]. There are no neuropathological criteria that allow to distinguish between PDD and DLB as this distinction can only be made based on clinical grounds; in PDD, EPS precede the onset of dementia by at least 12 months, whereas in DLB dementia is concomitant with or precedes EPS [96].

There are several neuropathological staging systems for LBD, the most frequently used are the Braak LBD stages and the Newcastle/McKeith criteria for LBD. Briefly, Braak LBD stages [14] postulate that nuclei in the medulla oblongata become initially affected (stage 1) and pathology spreads gradually to pons (stage 2), midbrain (stage 3), entorhinal cortex, and hippocampus (stage 4) and finally reaches the neocortex (stages 5 and 6). This topographical progression is not proposed in the Newcastle/McKeith criteria [96], which distinguish between brainstem predominant (PD), limbic (transitional; DLB), and diffuse neocortical (DLB) types of LBD. α-Syn aggregates in the form of LB do not show a continuous increase with increasing age. Instead, the degree and prevalence of Lewy body pathology started in the 41- to 60-year-old age group and then increased up 61–80 years of age. In older individuals, the prevalence of the lesions at the different stages decreased (Fig. 1f). This decrease of Lewy body pathology is unexpected and could be explained by a reduced life expectancy of Lewy body pathology patients compared with that of AD patients. However, confirmation in independent samples and further, more detailed studies are required to clarify these findings.

While the neuropathological hallmark lesions of LBDs are LB/LN other pathologies are highly prevalent in LBDs; Aβ depositions are present in up to 85% of LBD cases with dementia [61, 66, 76] and it has been suggested that striatal Aβ pathology is less frequent in PDD as compared to DLB (e.g., 18 vs. 100% in [76]), while in PD, Aβ pathology is in general less frequent (e.g., 55% in [66]) than in LBD with dementia. Similarly, τ pathology is frequently present in LBD [26, 66, 76] and a recently published study on 213 LBD cases with dementia found that 30% showed high Braak NFT stages V/VI [61]. Such cases which show neocortical LBD (i.e., DLB) and full-blown AD (i.e., Braak NFT stages V/VI) can be neuropathologically diagnosed as having mixed dementia (mixed AD/DLB), since the severity of either AD or LB pathology alone would be sufficient to represent a robust neuropathological correlate for clinical dementia. This raises the question as to why those cases clinically either present as DLB or AD. Using quantitative assessment of Aβ, τ-protein, and α-syn in such mixed AD/DLB we found that those who clinically presented with AD had higher τ loads than those with clinical LBD [156]. Moreover, the hierarchical distribution of τ-protein loads in clinical AD cases was similar to neuropathologically pure AD cases, while clinical DLB cases showed comparatively less hippocampal and temporal τ loads. These findings suggest that in clinical AD cases neuropathologically diagnosed as mixed AD/DLB neurodegeneration may be primarily driven by AD while LB pathology occurs later in the disease, possibly aggravated or even triggered by AD pathology. Conversely, in clinically diagnosed DLB cases, which show mixed AD/DLB at post-mortem examination, classical LB pathology (i.e., α-syn) may be the primary driver of the neurodegenerative process. While these data somehow suggest that in mixed cases the quantitatively predominant pathology determines the characteristic clinical pictures, a large clinico-pathological correlative study using conventional semi-quantitative methodology found in a subset of clinically diagnosed DLB cases only severe neocortical τ pathology and a complete lack of LB pathology [105]. Recently, Irwin and colleagues demonstrated that τ pathology in LBD with dementia independently predicted a shorter time interval between motor symptom and dementia onset as well as shorter overall survival [61], clearly indicating that—if present—τ plays a central role in the disease process of LBD and that the co-occurrence of both AD and DLB pathology is likely to have an aggravating effect on disease progression. Respective data from neuropathological post-mortem studies are corroborated by in vivo imaging studies showing higher amyloid and τ levels in LBD compared to controls [46, 47]. While AD pathology is the most important co-morbidity in LBD cases, they may also show other pathologies such as cerebrovascular disease to varying degrees of severity in up to 75% [67] and TDP-43 pathology in over 30% of cases [95]. More quantitative data on the amount of pathological burden in large clinico-pathological cohorts are needed to clarify the relative influence of Aβ, τ, α-syn, and TDP-43 on the clinical picture. This would pave the way for novel and more subtle clinico-pathological phenotypes, which, together with biomarkers, would allow to diagnose patients based on the actual underlying pathology.

Genetic data provide a tenuous link between synucleinopathies and tauopathies. H1 τ haplotype is associated with increased risk of PD [155], and has recently been associated with increased α-syn aggregation in DLB in a small post-mortem study [25]. There is also cross-talk between α-syn and τ in terms of disease-associated mutations. Mutations in or replications of the *SCNA* gene encoding α-syn that cause familial PD can also cause cognitive symptoms including dementia [104, 113, 126]. A recent genetic study indicates that the p.A152T variant of *MAPT* is likely associated with a higher risk of DLB [84]. Another potential genetic link between τ and α-syn is LRRK2. LRRK2 mutations usually give rise to α-syn pathology [52], but sometimes, they also cause tangle pathology. τ and α-syn pathology can also occur hand in hand without amyloid plaque pathology in rare cases, e.g., with hereditary spastic paraplegia due to SPG 7 mutations [148].

In addition to genetic data implying causative links between tauopathies and synucleinopathies, there are also multiple indications that pathological forms of τ and α-syn physically co-aggregate within neuronal cytoplasm [104]. Using double immunofluorescence labeling and mass-spectrometry, τ and α-syn have been observed in several studies to aggregate together in the same tangles, LB, and Lewy Neurites in PD and DLB brain [5, 24, 62]. Importantly, one recent study using novel conformation-specific antibodies demonstrated that toxic oligomeric forms of τ and α-syn accumulate together in PD and DLB [122]. In vitro, α-syn binds directly to τ via the microtubule-binding region of the τ protein and the C-terminus of α-syn [68]. This interaction is affected in a complex and as yet not completely understood manner by both phosphorylation and disease-associated mutations in the proteins [36, 104]. It is possible that τ and α-syn through their binding can cross-seed aggregation. Amyloidogenic proteins share some common characteristics in terms of their aggregation with a typically slow initial aggregation that becomes much faster after the process begins due to the new aggregates templating or seeding further aggregation [81]. This seeding process is not only relevant to the formation of large fibrils but also to the formation of oligomeric forms of proteins, which are likely the toxic species in the brain [81]. The seeding process is not necessarily limited to seeds made from the aggregating protein. Indeed, cross-seeding, in which “seeds”—some type of small aggregate—from one protein can induce aggregation of another protein—has been demonstrated across many different proteins. There is a small amount of evidence suggesting that τ and Aβ may cross-seed the formation of toxic aggregates in vitro [49]. Much more evidence implicates cross-seeding between α-syn and τ. In vitro, co-incubation of α-syn and τ synergistically promotes aggregation of both proteins [41], and in cultured neurons expressing FTDP-17 mutant τ, synthetic aggregates of α-syn seeded τ [50]. However, the cross-seeding of these proteins is not always reproduced, for example, using a sensitive FRET-based detection method, τ and α-syn did not cross-seed [55]. These conflicting results may be due to the ability of specific “strains” or confirmations of aggregates of the proteins, which induce the seeding, which has been observed for τ [22, 37], α-syn [111], and cross-seeding between them [50]. Similarly, distinct strains of Aβ have been observed, which induce different types of aggregates in vivo [133, 159]. Cross-seeding between Aβ and α-syn has been observed in vitro [109], along with hybrid oligomer formation [149]. Further work is warranted on understanding the cross-seeding process, and particularly on whether this causes the formation of toxic oligomeric species of the proteins.

Transgenic mouse models of tauopathies and synucleinopathies also support the idea of synergistic interactions of τ and α-syn in exacerbating neurodegenerative phenotypes. Overexpressing α-syn in transgenic mice causes τ phosphorylation [51, 160]. Combining pathologies in mice overexpressing fAD mutations in *APP* and *PSEN1*, FTDP-17 mutant *MAPT*, and PD associated *SCNA*, there is a synergistic effect of the pathological proteins resulting in exacerbated pathological and behavioral phenotypes [23]. Knocking out τ, however, does not prevent α-syn-related motor deficits in two models of PD, indicating that these proteins in addition to having synergistic effects can act independently to confer toxicity [103]. In wild-type mice injected with α-syn fibrils, both τ and TDP-43 aggregate in addition to α-syn [94]. There is also a substantial amount of evidence for cross-talk between Aβ and α-syn pathologies. NMR evidence shows molecular interactions between these proteins [91], and in a double transgenic mouse model, Aβ exacerbates α-syn accumulation and neuronal deficits [92]. Aβ induces phosphorylation of α-syn at Ser129 in vitro and pSer129 α-syn in brain tissue homogenates is related to the level of Aβ and Braak NFT stage [135]. Similar to Alzheimer’s disease, there is also evidence that synaptic degeneration is important in synucleinopathies with pathology associated with synaptic toxicity in vitro and in vivo [127, 154]. Less is known about molecular interactions of pathological proteins in synapse degeneration in synucleinopathies. τ reduction did not prevent motor deficits in α-syn overexpressing mice, indicating that τ is not necessary for synaptic deficits in this line [103].

## The frontotemporal lobar degeneration: amyotrophic lateral sclerosis (FTLD-ALS) spectrum and beyond

Both FTLD and ALS are heterogeneous diseases that can be sub-divided by their pathological lesions. Over the past few years, a substantial overlap has been observed in the pathologies, genetics, and clinical phenotypes of these complex diseases. FTLD can be subclassified in disorders that accumulate (1) τ, i.e., FTLD-tau, (2) TDP-43, i.e., FTLD-TDP, and (3) other FTLD-forms that accumulate other proteins (e.g., fused in sarcoma (FUS) etc.) [90]. FTLD-TDP and ALS share neuronal TDP-43 aggregates and a certain number of ALS cases also develop FTLD-TDP and vice versa [107].

FTLD-tau comprises several morphologically different tauopathies: Pick’s disease (PiD), corticobasal degeneration (CBD), progressive supranuclear palsy (PSP), argyrophilic grain disease (AGD), and neurofibrillary tangle-predominant dementia (NFTD) [90]. All these diseases have in common that τ is the sole protein that aggregates in these disorders. These diseases usually affect individuals below 70 years of age and concomitant AD-associated Aβ and τ as well as TDP-43 and LBD-associated α-syn may potentially be present but are only rarely seen [147] and hence not assumed to influence the disease process. Moreover, τ pathology in FTLD-tau is, except for NFTD, not restricted to neurons and neuronal processes. It also develops in astrocytes (astrocytic plaques in CBD, tufted astrocytes in PSP, τ-positive astrocytes in AGD) and oligodendrocytes (coiled bodies in AGD, CBD, PSP, PiD) [33]. Except for AGD, FTLD-tau disorders cause dementia and in most cases do not show co-pathologies [147]. AGD is usually present with low-level AD-related pathology and such cases do not show clinical dementia [72, 145]. Demented AGD cases, however, showed significantly more severe AD-related pathology (Aβ-plaques and NFTs) than non-demented AGD cases but less than in demented cases that had pure AD pathology, suggesting additive effects of AGD and AD pathology [72, 145]. τ pathology is also observed in some ALS cases, where it has been reported to correlate with cognitive decline [162].

FTLD-TDP is characterized by intracellular aggregates of phosphorylated TDP-43 in the neuronal cytoplasm, dendrites [107] as well as in oligodendroglial cells [106]. In FTLD-TDP and ALS cases, not only full length TDP-43 aggregates are found, but also truncated fragments, mainly C-terminal fragments of TDP-43, are also found [107]. In a subset of FTLD-TDP and ALS cases, hexanucleotide repeat expansions in the chromosome 9 open reading frame 72 (*C9ORF72*) have been seen. In these mutation carriers, dipeptide aggregates corresponding to abnormal translation of the pathological hexanucleotide repeats can be seen in the cytoplasm of neurons [101]. However, these aggregates do not correlate with disease severity or the coexisting TDP-43 pathology in the studies reported so far. The amount of TDP-43 pathology correlates more closely with the clinical severity of the disease in the studies conducted to date [89]. TDP-43 pathology in patients with the behavioral variant of FTLD-TDP (FTLD-TDP cases that show characteristic behavioral changes as key feature of the disease = bvFTLD-TDP) and with sporadic ALS show a characteristic pattern that suggests anatomical stages of TDP-43 pathology spreading that differed among bvFTLD-TDP and ALS [16, 18].

TDP-43 pathology is also found in AD cases [4, 153]. Recently, Josephs and colleagues reported that TDP-43 pathology in AD is seen in over 50% of cases where it spreads in a distinct pattern [70, 71], which was also observed in LBD and aged controls by others [95]. Moreover, colocalization of phosphorylated TDP-43 and τ-containing NFTs has been reported but it is not yet clear whether this finding is due to unspecific co-staining of NFTs with the pTDP-43 antibody or whether indeed phosphorylated TDP-43 accumulates in NFTs. A similar questionable colocalization of τ and TDP-43 is speculated in granulovacuolar degeneration (GVD) [75]. GVD has been shown to represent an AD-related lesion [139] exhibiting phosphorylated TDP-43 as well as numerous other proteins in neuronal cytoplasmic vacuoles including τ [34] and phosphorylated Aβ [83].

There is substantial overlap in the genetics of FTD and ALS [115]. Mutations in *C9ORF72*, *TARDBP, FUS, VCP, UBQLN2*, *SQSTM1*, and *CHMP2B* have all been associated with both ALS and FTD [46, 84], and mutations in *hgRNPA2B1* and can cause multisystem proteinopathy and ALS [80]. *TARDBP* mutations (TDP-43 gene) can lead to FTLD-TDP and/or rare ALS cases. Usually, no other proteins are aggregated in non-age-related amounts in these cases [74]. However, a case report shows a *TARDBP* mutation in a fAD case indicating a potential link from TDP-43 directly to AD [100]. *C9ORF72* mutations go in most instances along with TDP-43 pathology either with a FTLD-TDP or an ALS-related pattern [31, 116]. In single cases, AD-related pathology also occurred as relevant pathological lesion in *C9ORF72* mutation carriers, but the interpretation of this finding is controversial [19, 30, 54]. One of the most interesting aspects of *C9ORF72* mutations is that in families with a distinct *C9ORF72* mutation, the same mutation in some family members causes FTLD, whereas other family members with the same mutation developed ALS [20]. As such, other factors than the mutations appear to act as disease modifiers with impact on the primary vulnerability of distinct neurons. Since the C9orf72-related dipeptide aggregates do not correlate with the clinical symptomatology but with the TDP-43 pathology [107, 121], it is tempting to speculate that TDP-43-specific properties play a role in the primary targeting of the disease. In rare cases, mutations in the valosin-containing protein lead to FTLD-TDP or ALS [158]. Here, nuclear TDP-43-valosin-containing protein aggregates are the predominant lesion although cytoplasmic TDP-43 aggregates can also in different amounts [107, 121]. In this disorder, similar aggregates were found in skeletal muscle and in the myocardium [58, 158].

Pathological proteins typically associated with ALS and FTLD also show multiple mutual interactions including co-aggregation [10]. An interesting phenomenon emerging in the ALS protein aggregation literature is the role of low complexity domains in the RNA-binding proteins implicated in disease pathogenesis contributing to pathological protein aggregation. Low complexity domains mediate liquid–liquid phase separation which in healthy cells contributes to the assembly of membrane-less organelles such as RNA transport granules and stress granules; but in disease, the liquid phase transition initiates fibrillization of aggregates [99]. This may explain the co-aggregation of different low complexity domain-containing proteins such as TDP-43 and FUS [79]. Interestingly, dipeptide repeat proteins produced from unconventional non-ATG dependent translation of *C9ORF72* expansions, the most common cause of ALS and FTLD, have recently been found to interact with RNA-binding proteins with low complexity sequence domains in Drosophila [86]. This finding provides a potentially unifying mechanism for the disparate causes of ALS/FTLD converging on disrupted phase transitions that impair the assembly, dynamics, and function of membrane-less organelles that cause widespread cellular abnormalities including impairments in nucleolar function, nucleocytoplasmic transport, RNA splicing, translation, and abnormal protein aggregation.

## Discussion

Here, we have reviewed neuropathological and experimental findings, which together point to pathogenic links between the aggregation of Aβ, τ, TDP-43, and α-syn. These links are most evident in fAD cases with mutations in the *APP*, *PSEN1*, or *PSEN2* genes because these cases often show aggregates of all four proteins in the brain due to mutation-driven production of high amounts of Aβ. Even a larger number of sporadic AD cases exhibit Aβ, τ, and TDP-43 aggregates [4, 70, 153]. Given the fact that τ-aggregates develop in restricted areas of the brain before Aβ, TDP-43, and α-syn lesions occur, one could speculate that τ pathology initiates AD and the aggregation of other proteins is just a secondary phenomenon. However, most people will accumulate brainstem τ pathology (usually between 40 and 50 years of age) [15] but only 32% will develop symptomatic AD in their lifetimes [3], and genetic evidence strongly implicates changes in Aβ as crucial for initiating the disease process. Thus, τ pathology alone appears not to be sufficient to develop AD as defined according to the recommended criteria [60], Aβ pathology must also be involved. In other words, one can hypothesize that accumulation and aggregation of abnormal τ-protein aggregates is an age-related phenomenon, which becomes overtly pathogenic and allowed to spread throughout the brain in the presence of Aβ aggregates. Post-translationally modified forms of Aβ, such as Aβ_N3pE_ and _pSer8_Aβ, may play an important role in the maturation of these aggregates [117]. Thus, maturation of protein aggregates, especially Aβ aggregates, appears to play a major role in the development of the disease [117]. In animal models for Aβ pathology, Aβ pathology alone is not sufficient to develop the full spectrum of AD pathology [39, 56, 134], although it is possible that this is in part due to the lifespan of rodents and in part due to differences in rodent and human τ. Notwithstanding these caveats, the Aβ-expressing animal model findings do support our hypothesis that τ pathology is an essential prerequisite for the development of AD but only in combination with Aβ. τ-mutations in the absence of Aβ (in man and in transgenic mice) lead mainly to a non-AD tauopathy and only very rarely are associated with AD [27, 59]. Moreover, the AD pathology in APP mutation carriers develops in adulthood after a normal childhood, i.e., when τ pathology becomes more and more prevalent in the published autopsy cohorts meaning that once abnormal τ occurs, the disease starts to develop, implying that τ is a prerequisite for disease.

Interactions between Aβ and TDP-43 or α-syn may follow similar principles as that with τ. The AD-specific progression pattern of AD-related TDP-43 aggregates [70] is different from that in FTLD-TDP and ALS cases [16, 18], which argues in favor of the idea that Aβ also exaggerates AD-related TDP-43 pathology. An argument for the interaction between Aβ and α-syn aggregates may be the frequent coincidence of full-blown AD and LBD pathology in demented patients that were clinically diagnosed as LBD patients (PDD and/or DLB), as well as the detection of LBDs in fAD cases [88, 125]. Moreover, in the absence of Aβ, TARDBP and *SCNA* mutations do not cause AD but ALS or PD [74, 100, 113].

Other proteins (in addition to Aβ) may also have triggering effects for neurodegeneration. The most attractive candidates are dipeptide aggregates in C9ORF72 hexanucleotide repeat mutation carriers developing ALS or FTLD-TDP with TDP-43 aggregates [101]. Moreover, a case report also suggests that mutant paraplegin (*SPG7* mutation) triggers both brainstem τ and α-syn pathology in the absence of Aβ [148]. That one MAPT mutation causes α-syn changes in addition to τ pathology and that a mutation in LRRK2 can induce both, α-syn—and in a few cases τ—pathology [52, 84] further argues for a link between α-syn and τ.

Thus, several proteins can lead to neurodegeneration alone or in concert with one another. Intraneuronal and intracellular protein aggregates appear to be most relevant for the neurodegenerative process because they occur in all of the discussed neurodegenerative disorders and correlate well with cognitive decline and neuron loss [6, 45, 138]. It appears that the neuropathological type of neurodegenerative disorder simply reflects the circumstances of protein aggregation and that the pathogenic process of protein aggregation behind the phenotype has common principles in several neurodegenerative disorders, such as spreading of disease-related pathology [12, 14, 17, 143] and the involvement of post-translationally modified proteins in the disease-specific aggregates [38, 107, 140].

An argument for the hypothesis that the “phenotype of neurodegeneration” is the result of a critical accumulation of soluble and/or insoluble protein aggregates in neurons—regardless of the protein that aggregates—is provided by an artificial mouse model that produces intraneuronal Aβ aggregates after cleavage from an enkephalin-signal-peptide Aβ_42_ construct, which shows significant neuron loss and an ALS-like phenotype with serious motor dysfunction (APP48 mice) [1]. Under this artificial paradigm, Aβ and/or its soluble and/or insoluble aggregates show different neurodegenerative effects than under the more AD-related paradigm of APP overexpression [118]. However, post-translational modification of Aβ similar to that seen in AD cases also occurred in the intracellular Aβ_42_ aggregates in this artificial mouse model [118] indicating that post-translational modifications may play an important role for the neurodegeneration process in general. Thus, artificial expression of an aggregation-prone protein in neurons causes neurodegeneration similar to other intraneuronal proteins that develop aggregates such as τ or TDP-43, whereas mainly extracellular deposition of the same protein had only limited effects supporting the idea that the intracellular protein accumulation may be a prerequisite for neurodegeneration, whereas extracellular Aβ aggregates may catalyze spreading of intracellular aggregates while interacting and aggregating with the respective proteins.

**Fig. 3:**
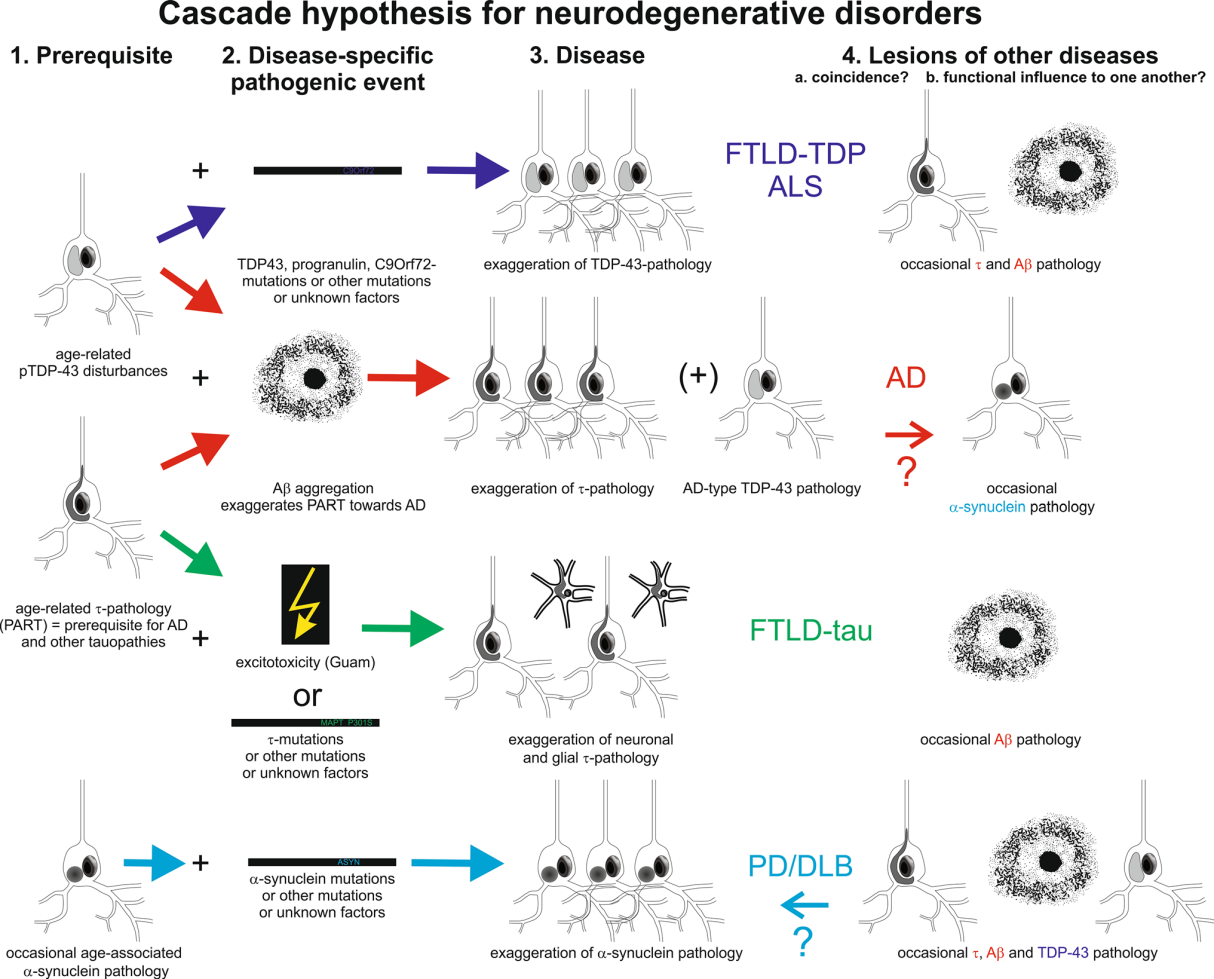
Cascade hypothesis for neurodegenerative disorders: Pathological protein interactions contribute to neurodegeneration. Schematic representation of the interplay between the different neurodegenerative protein aggregates and their related diseases. As a result of the current knowledge about disease progression and experimental evidence about protein-aggregate interactions in vivo an in vitro, we hypothesize that abnormal τ-protein accumulation is an event that happens during aging in the brain stem of nearly everyone above 40 years of age [15] (*1* prerequisite for disease). AD appears to develop when Aβ aggregates occur and initiate more pathological accumulation of τ and its spread through the brain (*2* disease-specific pathogenic event and *3* disease). Maturation of plaque-associated and soluble/dispersible Aβ-aggregates thereby appear to be a critical event in the progression of the disease. Abnormal τ-protein aggregates, TDP-43 aggregates, and α-syn aggregates occur in smaller subset of individuals during age as shown in Fig. 1 (it is not clear whether it is a result of aging or the early stages of neurodegeneration). Once Aβ aggregates prevail in a certain amount or biochemical maturation stage, it is tempting to speculate that these Aβ aggregates may also exaggerate/catalyze TDP-43 or possibly also α-syn pathology in pattern similar to that seen in AD or AD-LBD (*4* α-syn pathology is typical for PD/DLB and can be interpreted as pure coincidence or as influenced by/influencing AD pathology or its clinical picture). In the absence of Aβ and/or after exposure to other triggers (e.g., disease-specific mutations, excitotoxicity in Guam disease [35]) τ, α-syn, and TDP-43 aggregates develop other neurodegenerative disorders such as FTLD-tau, FTLD-TDP, ALS, or PD/DLB. *AD* Alzheimer’s disease, *FTLD* frontotemporal lobar degeneration, *ALS* amyotrophic lateral sclerosis (synonymous with MND—motor neuron disease), *LBD* Lewy body disease [including Parkinson’s disease (PD) and DLB]

The above-mentioned considerations argue against the classical view of the amyloid cascade hypothesis insofar as Aβ does not seem to the one and only player that causes all the rest of AD pathology. Amyloid as a key factor in AD pathogenesis is strongly supported, particularly by genetics of fAD, but it is also clear that alone, amyloid pathology is insufficient to drive neurodegeneration. The weight of the evidence on mechanisms of neuron loss in AD sides with τ being the driving toxic force. The data we have summarized here on co-occurrence of amyloid and τ-pathologies in human brain and their mechanistic interactions in model systems support the intriguing possibility that in sporadic AD, age-related τ-accumulation as observed in PART occurs earlier and independently from Aβ, thus representing a prerequisite for Aβ to kick off the pathogenesis of AD. In the event that both proteins in an aggregated form co-occur in the brain, they appear to become particularly toxic and presumably interact to lead to the devastating neurodegeneration in AD. Lesions typical for other neurodegenerative disorders or vascular lesions may co-exist and either interact with the AD-related protein aggregates or producing brain lesions that are added to the AD-related neurodegeneration. Other tauopathies, TDP-43 proteinopathies, and synucleinopathies may follow a similar cascade of prerequisite lesion plus secondary disease-specific pathogenic event as depicted in Fig. 3.

One critical point in this cascade hypothesis is the point that mutation-driven disease might need a prerequisite lesion. One can argue that the mutations will drive even the initial lesions. However, if this would be the case, mutation-driven neurodegenerative disorders should manifest earlier in life during childhood because the mutant genes are expressed even at that age. However, most frequently, these disorders manifest usually in the 4th–6th decade of life or later [29, 42, 59, 129, 130, 137]. Accordingly, an age-related event seems to be required before the mutant protein can set a disease-specific pathogenic event. This concept is to some degree in line with the two-hit concept for TDP-43-proteinopathies [112].

The accumulation of misfolded proteins in the human brain is clearly associated with advanced age. However, the underlying mechanisms of this age-associated phenomenon are still unclear and the respective role of, e.g., DNA damage, autophagy impairment, cellular senescence, oxidative stress, and mitochondrial dysfunction are yet to be elucidated. Such elucidation may also provide further insights into cerebral multimorbidity as the age-associated impairment of underlying mechanisms to maintain cellular homeostasis may vary between individuals, possibly based on their respective genetic background. For example, GWAS studies point toward a role of disturbed autophagic/lysosomal function in DLB but not in PDD, while the opposite was suggested for mitochondrial dysfunction. Therefore, the pathomechanisms of cerebral multimorbidity may not only be explained by protein–protein interactions but also by age-associated failure of basic cellular mechanisms, which may show individual variations.

The presence of multiple protein aggregates in patients with neurodegenerative symptoms indicates that in many cases not only one disease, such as AD is responsible for the clinical symptoms but that secondary components such as TDP-43 inclusions or even vascular lesions contribute to the development of the symptoms of a given patient as well. Thus, diagnostic and therapeutic regimes must not only focus on the most evident pathology such as AD but also need to take into account other neurodegenerative and vascular lesions. Thus, for the clinical diagnostic procedure, it appears to be essential to screen not only for AD lesions, but also for α-syn, TDP-43, and for vascular lesions.

## Conclusion

τ, TDP-43, and α-syn alone lead to specific neurodegenerative disorders that do not necessarily show multiple proteins aggregating in the respective disorders such as in FTLD-tau, FTLD-TDP, ALS, and PD, whereas AD appears to have at least a two-step pathogenesis with alterations in Aβ likely initiating the actual disease process, but with τ accumulation and spread through the brain being the essential step to cause disease symptoms. It is possible that pathological accumulations of τ independent of Aβ are a prerequisite for AD with extracellular Aβ being the second step that exaggerates or catalyzes the pathological process leading to clinical dementia. Similarly, TDP-43 and α-syn pathologies probably develop independently (and can cause clinical disease) but extracellular Aβ may aggravate or catalyze the spread of these pathologies throughout the brain. Accumulating evidence suggests that the fibrillar aggregates of all of these proteins are not likely directly toxic to neurons but reflect—and in some cases exacerbate—the accumulation of toxic soluble forms of the proteins.

Data from human autopsy material and experimental model systems indicate multiple interactions of pathological proteins both directly in terms of co-aggregation and indirectly, for example, involvement in the same molecular pathways to neurodegeneration. Recent data also make it tempting to speculate that pathological proteins interact in the process of spread of the disease through the brain. However, our knowledge on cerebral multimorbidity is still limited as this multimorbidity shows considerable qualitative and quantitative heterogeneity between cases, and hence large-scale studies on human post-mortem brains, which combine both detailed clinical data and quantitative data on the amount of protein-aggregate burden, are needed to further our understanding of protein–protein interactions in the multimorbid old brain. Understanding these interactions will be important to develop effective therapeutic targets to reduce toxicity and possibly to stop these diseases in their tracks (Fig. 3).
